# Directions and Perspectives for Preventive Activities in Primary Care—Patients’ Health-Promoting and Health-Risk Behaviours

**DOI:** 10.3390/nu18020346

**Published:** 2026-01-21

**Authors:** Anna Domańska, Sabina Lachowicz-Wiśniewska, Wioletta Żukiewicz-Sobczak

**Affiliations:** 1 Department of Nutrition and Food, The Faculty of Medicine and Health Science, University of Kalisz, W. Bogusławskiego 2, 62-800 Kalisz, Poland; anna_domanska@onet.pl; 2 Department of Biotechnology and Food Analysis, The Faculty of Production Engineering, Wrocław University of Economics and Business, Komandorska 118/120, 53-345 Wrocław, Poland; 3 Department of Biological Bases of Food and Feed Technologies, The Faculty of Production Engineering, University of Life Sciences in Lublin, Głęboka 28, 20-612 Lublin, Poland

**Keywords:** primary care, cardiovascular risk, metabolic syndrome, obesity, health behaviours, prevention, SCORE2

## Abstract

Non-communicable diseases, particularly cardiovascular diseases (CVD) and metabolic syndrome (MS), remain a major challenge for primary health care (PHC). This study aimed to assess cardiometabolic risk and health behaviours in adult PHC patients using routine preventive screening. This prospective observational study included 506 adults attending routine consultations in an urban PHC centre in Poland. Preventive assessment included anthropometric measurements (body weight, height, BMI, and waist circumference), blood pressure, lipid profile, and fasting glucose levels. Health behaviours were recorded using the standardised NFZ CHUK questionnaire. The 10-year CVD risk was estimated using the SCORE2 algorithm. Multivariable logistic regression was used to identify independent factors associated with high cardiovascular risk (SCORE2 ≥ 5%) and of a composite endpoint defined as the presence of any non-optimal biochemical parameter. Nearly half of the participants had excess body weight (overweight or obesity), and more than half met criteria for central obesity. Borderline or elevated total cholesterol was found in 47% of patients, abnormal LDL in 27%, low HDL-C (<40 mg/dL) in 80% (84% when applying sex-specific cut-offs), and impaired fasting glucose or diabetes in about 12%. High SCORE2 risk (≥5%) was observed in approximately 9% of the cohort. In multivariable models, SCORE2 components (age, sex, and smoking) were, as expected, associated with high SCORE2 risk, and obesity (BMI ≥ 30 kg/m^2^)—a factor not included in SCORE2—was additionally associated with higher risk. Additionally, age, male sex, and obesity also predicted the presence of at least one non-optimal biochemical marker. The prevalence of high SCORE2 risk increased from 1.2% in patients with 0–1 modifiable risk factor to 25.7% in those with 4–5 factors. Lower educational attainment was associated with a higher proportion of high-risk individuals in univariate analysis. Routine preventive activities in PHC enable the identification of important lipid and glucose abnormalities and the clustering of modifiable risk factors, even in a relatively young, highly educated population. Systematic cardiovascular screening and a focus on patients with accumulated risk factors should remain a priority in PHC to enable early identification of high-risk patients and timely implementation of lifestyle and therapeutic interventions.

## 1. Introduction

Cardiovascular diseases and other chronic non-communicable diseases remain the leading cause of premature mortality worldwide, despite the increase in life expectancy observed over recent decades. A substantial proportion of these conditions—including stroke, myocardial infarction, type 2 diabetes, selected cancers, and chronic respiratory diseases—are associated with modifiable risk factors such as diet, level of physical activity, tobacco smoking, obesity, and disturbances in lipid and carbohydrate metabolism. The occurrence, severity, and consequences of these diseases can be largely reduced through preventive actions implemented at different levels—from primordial prevention, targeting the social determinants of health, through primary, secondary, and tertiary prevention, to quaternary prevention aimed at protecting patients from overmedicalisation [1]. Despite robust scientific evidence demonstrating the cost-effectiveness and efficacy of preventive measures, their implementation in clinical practice remains insufficient in many countries, and a substantial proportion of patients do not receive all recommended preventive services [1].

In this context, primary health care (PHC) constitutes a key component of the health care system, linking individual treatment with a population-based approach. According to the Polish Act on Primary Health Care [2], PHC provides comprehensive and coordinated services at the patient’s place of residence, including, among others, identifying health needs, establishing health priorities, and implementing preventive measures related to lifestyle diseases. Similar principles have long underpinned the concept of community-oriented primary care (COPC), which integrates individual patient care with epidemiologically informed community-level activities. Experiences from COPC programmes suggest that this approach can lead to significant improvements in population health indicators, provided that PHC teams are adequately prepared, epidemiological tools are used effectively, and close interdisciplinary collaboration is ensured [3].

A key dimension of prevention in PHC is patients’ health behaviours. Research on multiple health behaviour change has shown that tendencies to engage in specific health-promoting activities (e.g., physical activity, healthy eating) and health-risk behaviours (e.g., smoking, excessive alcohol consumption) frequently co-occur and form recognisable lifestyle patterns [4]. Populations exposed to multiple risk factors are particularly vulnerable to the development of chronic non-communicable diseases and generate disproportionately high health care costs. Therefore, understanding the coexistence of health-promoting and health-risk behaviours and their associations with clinical parameters is crucial for designing effective interventions [4]. At the same time, studies on multiple behaviour change suggest that modifying one domain (e.g., increasing physical activity) may facilitate favourable changes in others (e.g., improving diet quality), which opens up opportunities to design interventions that exploit “transfer effects” between behaviours [4].

However, translating this concept into PHC practice requires not only the use of screening tools but also high-quality health counselling. Qualitative studies among primary care nurses involved in cardiovascular prevention programmes have shown that health-promoting dialogues are complex processes that require balancing support and pressure, tailoring the conversation to patients’ needs while still guiding it, and building trust while, at times, appealing to fear [5]. Primary care physicians, in turn, emphasize the importance of continuity of care and long-term doctor–patient relationships for effective prevention; yet, in everyday practice, they are often forced to deprioritize preventive activities due to time constraints, urgent clinical problems, and the large number of preventive recommendations included in guidelines [6]. Reviews of the literature on the implementation of different levels of prevention in clinical practice also identify numerous systemic barriers, such as provider overload, insufficient integration of health promotion with routine care, and socioeconomic inequalities that limit access to preventive services [1].

Nutrition is a central, modifiable determinant of cardiometabolic risk and a major driver of interindividual variability in routinely assessed markers used in primary care prevention (blood pressure, lipid profile, fasting glucose, BMI, and waist circumference). Current prevention frameworks emphasize that, alongside smoking cessation and physical activity, dietary counseling should be considered a first-line strategy for improving risk-factor control, because even relatively small habitual changes (e.g., reduced sodium and saturated fat intake, higher fiber density) can translate into clinically meaningful shifts in these biomarkers at the population level [7,8,9].

The relationship between diet and the measurements applied in this study is biologically plausible and supported by intervention evidence. Sodium reduction and dietary patterns rich in potassium, magnesium, and plant foods lower blood pressure. Conversely, replacing saturated fats with unsaturated fats reduces LDL-C and improves overall lipid profile. High intake of refined carbohydrates and added sugars tends to increase triglycerides and worsen glycemic control. Notably, the DASH dietary pattern has been consistently associated with reductions in both systolic and diastolic blood pressure. Dietary approaches focusing on cardioprotective fats and fibre-rich foods have demonstrated LDL-lowering effects comparable to low-intensity pharmacotherapy in selected populations [9,10,11,12].

Beyond single nutrients, whole-diet patterns appear especially relevant in contemporary primary care because they capture habitual food choices, energy density, and the level of food processing. Mediterranean-style eating has been associated with favorable changes in central adiposity and multiple components of metabolic syndrome (waist circumference, fasting glucose, triglycerides, and HDL-C), whereas higher consumption of ultra-processed foods and sugar-sweetened beverages is linked to greater cardiometabolic risk and an increased incidence of cardiovascular disease and type 2 diabetes. Therefore, considering the nutritional context is important when interpreting anthropometric and biochemical findings and when translating screening results into practical prevention pathways in PHC [13,14,15,16,17].

In line with current cardiology guidelines, including the recommendations of the European Society of Cardiology, primary health care is the setting in which individuals at increased cardiovascular risk should be systematically identified using standardized risk assessment tools (e.g., SCORE2), blood pressure measurements, evaluation of biochemical parameters, and basic anthropometric indices. In Poland, this function is supported by the CHUK preventive examination card of the National Health Fund, which includes a lifestyle interview (tobacco smoking, diet, and physical activity), an assessment of anthropometric indicators (BMI and waist circumference), and blood pressure measurement. Despite the introduction of such solutions within PHC contractual services, there is still a lack of data describing how often and to what extent, in everyday practice, comprehensive assessment of patients’ health behaviours is performed and how these behaviours are related to metabolic profile and estimated cardiovascular risk in adult patients attending routine primary care consultations.

Therefore, the aim of the present study was to perform cardiovascular risk screening in a group of patients attending routine primary health care consultations and to analyze health-promoting and health-risk behaviors in relation to anthropometric and biochemical parameters. Specifically, we assessed the coexistence of excess body weight and abdominal obesity, disturbances in lipid and carbohydrate metabolism, and behaviours such as tobacco smoking and insufficient physical activity, and their associations with the estimated 10-year risk of cardiovascular events, as determined by the SCORE2 algorithm, in a population of primary care patients. The findings aim to provide a basis for defining practical directions to intensify preventive activities in primary health care, focusing on multidimensional lifestyle modification and the early identification of individuals who require more advanced diagnostics and specialist care.

## 2. Materials and Methods

The study employed a prospective observational design, in accordance with current recommendations for research in primary health care settings [18,19]. It was conducted as part of the project “Assessment of health-promoting and health-risk behaviours in a selected group of patients. Evaluation of peripheral blood morphology parameters and selected vitamins in patients with selected nervous system and cardiovascular disorders” (Resolution No. 5/2021), between 1 December 2021 and 1 December 2022 at the Primed Medical Centre in Lublin, Poland. The study was embedded in routine primary care practice and did not interfere with the standard provision of health care services.

### 2.1. Study Population

A total of 506 patients attending primary care for routine consultations were enrolled in the study, having provided informed consent to participate. The primary inclusion criterion was the patient’s voluntary consent, as outlined in the study protocol. Within this prospective observational framework, the risk of cardiovascular disease was assessed in patients seeking medical advice in primary care. All assessments and measurements were performed in conjunction with standard diagnostic and therapeutic procedures, without disrupting routine care. Patients who agreed to participate were consecutively included in the study. Exclusion criteria were lack of informed consent, pregnancy, acute conditions requiring urgent medical intervention, and inability to complete the full set of planned laboratory tests and measurements.

### 2.2. Questionnaire

The study was conducted using a standardised questionnaire—the NFZ cardiovascular disease prevention form (“Karta badania profilaktycznego CHUK”, National Health Fund [20]). This official form (Annex No. 4 to Ordinance No. 69/2007/DSOZ of the President of the NFZ) includes patient identification data, a structured interview on risk factors (smoking, diet, physical activity), measurements (blood pressure, BMI, waist circumference), as well as an assessment of cardiovascular risk and recommendations for further management.

### 2.3. Scope of the Assessment

(i)  Anthropometric measurements: body weight (kg), height (cm), BMI, and body circumferences (arm, waist). Measurements were performed in accordance with NHANES guidelines [21] and the IAS/ICCR consensus on central obesity [22].
(ii)  Biochemical parameters: lipid profile (total cholesterol, HDL, LDL, triglycerides) and fasting glucose. Analyses were carried out according to ADA and ADLM recommendations [23,24].
(iii)  Physical examination: measurement of arterial blood pressure.
(iv)  Standardised interview (NFZ): assessment of health behaviours and cardiovascular risk factors.

BMI was classified according to WHO criteria as follows: underweight (<18.5 kg/m^2^), normal weight (18.5–24.9 kg/m^2^), overweight (25.0–29.9 kg/m^2^), obesity class I (30.0–34.9 kg/m^2^), obesity class II (35.0–39.9 kg/m^2^) and obesity class III (≥40.0 kg/m^2^). Waist circumference was categorised according to the IAS/ICCR consensus, using cut-off points of >80 cm for women and >95 cm for men to define central obesity [21]. Age was grouped into three categories: ≤50 years, 55–60 years, and >60 years, in line with the age distribution of the study sample. Cardiovascular risk was classified into the following categories: none, <1%, 1–5%, 5–9%, 10–15% and >15%, reflecting the categories used in clinical practice when interpreting SCORE2 results [25,26,27].

### 2.4. Procedure

(i)  Anthropometric measurements were performed by medical staff (nurses).
(ii)  Laboratory analyses were carried out and validated by certified laboratory diagnosticians.
(iii)  The results were interpreted by a family medicine physician.
(iv)  The risk of cardiovascular events was assessed using the SCORE2 algorithm, recommended by the European Society of Cardiology [26], taking into account age, sex, blood pressure values, cholesterol concentration, and smoking status [26,27].

Although patient enrolment was prospective and embedded in routine care, the present analyses are cross-sectional and based on a single preventive screening visit; therefore, incident cardiovascular events were not assessed.

### 2.5. Clinical Management

Based on the obtained results, patients received individual recommendations regarding health promotion and disease prevention. If necessary, they were referred for: (i) health education, (ii) further diagnostics and treatment in specialist outpatient clinics, and (iii) follow-up within primary health care.

### 2.6. Statistical Methods

The Shapiro–Wilk test was used to assess the normality of distribution. Due to the predominantly non-parametric distribution of continuous variables, between-group comparisons were performed using the Kruskal–Walli’s test. Categorical variables were compared using the chi-square test.

In addition, multivariable logistic regression models were constructed to identify independent determinants of (i) high cardiovascular risk (SCORE2 ≥ 5%) and (ii) the presence of any non-optimal biochemical parameter. High SCORE2 risk was defined as a 10-year cardiovascular risk category of 5–9%, 10–15% or >15%, according to the SCORE2 algorithm. The composite biochemical endpoint (“any non-optimal biochemical parameter”) was defined as the presence of at least one of the following: borderline or high total cholesterol (≥201 mg/dL), borderline or high LDL cholesterol (≥136 mg/dL), low HDL cholesterol (<40 mg/dL), borderline or high triglycerides (≥151 mg/dL), or impaired fasting glucose/diabetes (≥100 mg/dL).

For both models, independent variables included sex (male vs. female), age category (≥50 vs. <50 years), obesity (BMI ≥ 30 kg/m^2^ vs. <30 kg/m^2^), current smoking (yes vs. no), and physical activity (≥1 time/week vs. none). Family history of myocardial infarction or stroke (yes vs. no) was additionally included in the model for high SCORE2 risk. Odds ratios (ORs) with 95% confidence intervals (CIs) were calculated. A *p*-value < 0.05 was considered statistically significant. All analyses were performed using STATISTICA 13.3 PL (StatSoft Inc., Tulsa, OK, USA).

## 3. Results

### 3.1. Characteristics of the Study Population

The study was conducted among 506 patients attending primary care, of whom 37% were women and 63% were men (*p =* 0.006). Most participants were ≤50 years of age (79%), 17% were aged 55–60 years, and only 3% were over 60 years, indicating a predominance of individuals of working age in the analysed group.

With regard to nutritional status (BMI), underweight was found in 1.2% of respondents, normal body weight in 56%, while overweight and various degrees of obesity were observed in a total of approximately 42.7% of patients (overweight 31.6%, obesity class I 8.5%, class II 1.8%, class III 0.8%; *p =* 0.3519). This means that nearly every second participant had excess body weight despite the relatively young age structure. The problem of excess adiposity was even more pronounced when waist circumference was considered: increased waist circumference was found in about 53% of the sample overall (women > 80 cm—34%, men > 95 cm—19%; *p =* 0.001), indicating a high prevalence of central obesity in this population.

In terms of education, the majority of patients had higher education (79%), whereas 18% had secondary education and 1.5% each had vocational or incomplete higher education (*p =* 0.001). Regarding occupational status, 18% of respondents reported white-collar work, 4.4% manual work, 1.8% were retirees or disability pensioners, while as many as 32.4% were classified as “other” (*p =* 0.002), which may include, for example, students, homemakers, or individuals engaged in non-standard forms of employment.

Physical activity was reported by 48.6% of patients, while 51.4% admitted to not being physically active (*p =* 0.137). Thus, more than half of the studied population did not engage in regular physical activity despite a relatively high level of education. With respect to smoking, 82% of respondents declared that they did not smoke, while around 18% reported current smoking, most often having initiated the habit between 10 and 20 years of age (*p =* 0.041). At the same time, 65% of participants clearly disapproved of smoking (i.e., considered it harmful), whereas 35% did not explicitly disapprove of smoking (*p =* 0.045), which may indicate persisting more liberal attitudes towards this habit in a substantial subgroup.

In summary, education, type of work, sex, and smoking-related behaviours were significantly associated with age in the study population, whereas the distribution of BMI categories and self-reported physical activity did not differ significantly between age groups (Table 1).

### 3.2. Biochemical Profile and Estimated Cardiovascular Risk

The next stage of the study involved detailed diagnostics for cardiovascular diseases (CVD). Total cholesterol within the normal range (≤200 mg/dL) was found in 53% of patients, borderline values (200–250 mg/dL) in 41%, and elevated values (>250 mg/dL) in 6% of the study group (*p =* 0.048). Normal LDL cholesterol values (≤135 mg/dL) were observed in 73% of patients, borderline values (135–150 mg/dL) in 13.6%, and abnormal values (>150 mg/dL) in a further 13.6% (*p =* 0.004). For HDL cholesterol, minimum desirable levels (40 mg/dL in men and 50 mg/dL in women) were recorded in 6% and 10% of patients, respectively, whereas HDL-C < 40 mg/dL was found in 80% of the study population (*p* = 0.121). Importantly, when applying sex-specific thresholds for low HDL-C (<40 mg/dL in men and <50 mg/dL in women), low HDL-C was present in 84% of participants and was more common in men than in women (90.9% vs. 72.2%).

Triglyceride concentrations within the normal range (≤150 mg/dL) were noted in 80% of patients, borderline values (150–199 mg/dL) in 11.5%, and elevated values (≥200 mg/dL) in 8.5% (*p =* 0.003). Normal fasting glucose levels (≤99 mg/dL) were observed in 88% of participants, impaired fasting glucose indicative of prediabetes (100–125 mg/dL) in 11.3%, and values suggestive of diabetes (>125 mg/dL) in 0.6% of patients (*p =* 0.003).

Based on the medical interview, a positive family history of myocardial infarction or stroke was reported by 6.5% of patients, while 93.5% denied such events in first-degree relatives (*p =* 0.229. Data on estimated cardiovascular risk showed that 18% of patients had no risk, 30% had a risk < 1%, 43% had a risk of 1–5%, 7% had a risk of 5–9%, 2.5% had a risk of 10–15%, and 0.5% had a risk > 15% (*p =* 0.001). Blood pressure measurements were categorized into three groups: 120–129/80–84 mmHg in 57% of patients, 130–139/85–89 mmHg in 30%, and ≥140/90 mmHg in 13% (*p =* 0.001).

Overall, approximately 47% of patients had borderline or elevated total cholesterol levels, and nearly 27% showed abnormal LDL concentrations. Borderline or elevated triglycerides were present in approximately 20% of participants, while about 80% had reduced HDL levels, further deteriorating their lipid profile. Impaired glucose regulation (prediabetes or diabetes) was identified in approximately 12% of the study group. More than half of the patients (approximately 52%) had an estimated 10-year cardiovascular risk of ≥1%, and in about 9%, this risk was moderate or higher (≥5%). Additionally, in approximately 43% of patients, blood pressure values exceeded 130/85 mmHg. Age significantly differentiated total cholesterol, LDL, triglycerides, glucose levels, and estimated cardiovascular risk (*p* < 0.05; Table 2).

### 3.3. Associations Between BMI and Cardiometabolic Risk Markers

A statistically significant association was observed between BMI and total cholesterol, LDL, HDL, triglycerides, glucose levels, and estimated cardiovascular risk (*p =* 0.008 for total cholesterol; *p =* 0.001 for LDL, HDL, and triglycerides; *p =* 0.002 for glucose; Table 3). This indicates that with increasing BMI, an unfavourable, atherogenic lipid profile (higher LDL and triglyceride levels and lower HDL) became more frequent, as did abnormal fasting glycaemia, which is consistent with the typical picture of metabolic syndrome. These relationships are consistent with the proportions of patients with abnormal total cholesterol (6%), LDL (13.4%), triglycerides (8.5%), and prediabetes or diabetes (approximately 12% in total), as shown in Table 2. Importantly, BMI was not associated with a family history of myocardial infarction or stroke (*p =* 0.7075), suggesting that, in this cohort, current nutritional status and related modifiable risk factors may be more important determinants of the cardiovascular risk profile than hereditary burden alone (Table 3).

### 3.4. Multivariable Factors Associated with High Cardiovascular Risk and Non-Optimal Biochemical Profile

In multivariable logistic regression analysis, several factors were independently associated with high cardiovascular risk (SCORE2 ≥ 5%) (Table 4). After adjustment for all covariates, age ≥ 50 years was the strongest factor associated with high SCORE2 risk (OR = 29.02, 95% CI: 11.73–71.79; *p* < 0.001). Male sex was also significantly associated with increased odds of high cardiovascular risk compared with female sex (OR = 3.89, 95% CI: 1.76–8.61; *p* < 0.001). Obesity (BMI ≥ 30 kg/m^2^) was associated with a higher SCORE2 risk (OR = 4.56, 95% CI: 1.80–11.55; *p* = 0.001), as was current smoking (OR = 7.16, 95% CI: 3.05–16.82; *p* < 0.001). In contrast, physical activity (≥1 time/week) and family history of myocardial infarction or stroke did not reach statistical significance in the fully adjusted model (*p* > 0.05).

In a second multivariable model including a composite endpoint of any non-optimal biochemical parameter (total cholesterol, LDL cholesterol, HDL cholesterol, triglycerides, or fasting glucose), male sex, older age, and obesity were independently associated with an adverse metabolic profile (Table 5). Participants aged ≥ 50 years had a more than threefold higher likelihood of presenting at least one non-optimal biochemical marker than those < 50 years (OR = 3.35, 95% CI: 2.01–5.59; *p* < 0.001). Male sex was associated with approximately twofold higher odds of having at least one abnormal or borderline biochemical parameter (OR = 2.08, 95% CI: 1.39–3.12; *p* < 0.001), and obesity (BMI ≥ 30 kg/m^2^) was also a significantly associated with (OR = 2.16, 95% CI: 1.10–4.28; *p* = 0.026). Current smoking and physical activity were not significantly associated with the composite biochemical endpoint in the adjusted model (*p* > 0.05).

Together, these models indicate that older age, male sex, obesity, and current smoking are the key factors driving elevated 10-year cardiovascular risk in this primary care population, while age, sex, and obesity are also strongly linked to the presence of at least one adverse biochemical marker.

When clustering of modifiable risk factors was considered (overweight/obesity, elevated blood pressure, current smoking, physical inactivity, and any non-optimal biochemical parameter), most patients presented with multiple coexisting risk factors (Table 6). Only 10.5% of patients had no risk factors, and 23.4% had one, whereas 30.5% had two, 21.0% had three, 12.1% had four, and 2.6% had all five risk factors. The prevalence of high SCORE2 risk (≥5%) increased markedly with the number of coexisting risk factors, from 1.2% in patients with 0–1 factor, through 9.2% in those with 2–3 factors, to 25.7% in those with 4–5 factors (*p* < 0.001), indicating a clear dose–response relationship between risk factor clustering and estimated 10-year cardiovascular risk.

In univariate analysis, educational level was also associated with cardiovascular risk (Table 7). Patients with less than tertiary education had a higher prevalence of high SCORE2 risk (≥5%) compared with those with tertiary education (17.5% vs. 6.9%; *p* = 0.002). This pattern suggests that lower educational attainment may be linked to an unfavourable cardiovascular risk profile in primary care populations, although the association was attenuated after adjustment for classical risk factors in multivariable models.

## 4. Discussion

Monitoring health behaviors, along with anthropometric and biochemical parameters, is the cornerstone of modern cardiovascular prevention. Epidemiological data indicate that the burden of non-communicable diseases has been increasing over recent decades, with metabolic syndrome (MS) representing a cluster of risk factors that predict the development of cardiovascular disease (CVD) and type 2 diabetes [28,29,30]. Early identification of MS enables timely intervention targeting modifiable cardiometabolic risk factors. The World Health Organization and other agencies highlight that risk factors related to CVD and MS are central in the prevention and treatment of cardiometabolic disorders [31,32]. The WHO’s “Global Action Plan for the Prevention and Control of Noncommunicable Diseases 2013–2020” emphasizes the need to reduce physical inactivity and prevent diabetes, obesity, MS, and CVD through population-wide strategies [32,33,34]. National reports from Poland similarly indicate a persistently high burden of cardiometabolic risk factors in adults, including obesity, hypertension, and dyslipidaemia, placing the country among European settings with substantial cardiovascular mortality. Against this background, data from real-world primary care populations are particularly important for tailoring effective prevention strategies.

The present study of 506 primary care patients confirms significant associations between anthropometric indicators, lifestyle behaviours, and biochemical markers of cardiovascular risk. Approximately one third of participants were overweight, and about 11% were obese (I–III degree), while more than half fulfilled criteria for central obesity based on waist circumference. Elevated BMI was significantly associated with abnormal levels of total cholesterol, LDL, triglycerides, and fasting glucose, as well as with estimated cardiovascular risk. This pattern is consistent with large-scale population studies, which show that higher BMI is strongly associated with dyslipidemia and impaired glucose metabolism [35]. Recent evidence also suggests that BMI variability itself may independently predict cardiovascular events beyond mean BMI values [36]. Notably, these unfavourable cardiometabolic profiles were observed in a relatively young, predominantly highly educated sample, which could be expected to have a lower baseline risk. This emphasizes that traditional protective factors, such as higher education, do not fully offset the impact of adverse lifestyle patterns and excess adiposity.

Waist circumference, used in our study as a proxy for central adiposity, further highlighted sex-specific accumulation of abdominal fat. Central obesity is increasingly recognised as a better predictor of cardiometabolic risk than BMI alone, as visceral fat is more metabolically active and pro-inflammatory. Previous research has shown that waist circumference and waist-to-height ratio outperform BMI in predicting future CVD and mortality [37]. Our findings, indicating a high prevalence of increased waist circumference among a relatively young, highly educated population, support the recommendation to routinely monitor waist circumference in primary care, in addition to BMI. In addition, the simultaneous assessment of waist circumference, BMI, lipid profile, fasting glucose, and estimated SCORE2 risk in our study reflects a comprehensive “real-world” screening approach that goes beyond minimal standard care and may serve as a practical model for cardiovascular prevention in Polish primary care.

Smoking emerged as a statistically significant factor associated with cardiovascular risk. Although the overall prevalence of smoking was moderate, a substantial proportion of smokers reported initiation of tobacco use in adolescence, which is in line with NHANES analyses indicating that smoking amplifies the impact of other risk factors and leads to higher global CVD risk [38,39]. In our multivariable model, current smoking was associated with more than a sevenfold increase in the odds of high 10-year cardiovascular risk (SCORE2 ≥ 5%) (OR ≈ 7.2), even after adjustment for age, sex, obesity, and other covariates. In contrast, only 48.6% of participants reported regular physical activity, reflecting global trends of insufficient exercise. Data from the European Society of Cardiology and meta-analyses indicate that regular aerobic and combined physical activity can significantly improve MS components and reduce cardiovascular risk by up to one-quarter [30,34,38]. In our sample, the coexistence of excess body weight, central obesity, dyslipidaemia, impaired fasting glucose, smoking, and low physical activity points to an unfavourable cardiometabolic profile compatible with MS and reinforces the need for multifactorial interventions rather than single-risk-factor approaches.

An interesting aspect of our findings is the role of socioeconomic factors. The majority of participants (79%) reported having higher education; however, patients with less than tertiary education had a significantly higher prevalence of high SCORE2 risk (17.5% vs. 6.9% in those with tertiary education). This is consistent with international evidence showing that lower socioeconomic status and lower educational attainment are linked to higher incidence and mortality from CVD [40]. However, even in populations with relatively high levels of education, an apparent gap persists between knowledge and behavior. Our data, demonstrating a high prevalence of modifiable risk factors in a predominantly well-educated group, support the notion that health literacy alone is insufficient without effective behavioural interventions and structural support. This also suggests that education-related differences in cardiovascular risk may be largely mediated by differences in exposure to classical risk factors (age, obesity, smoking, physical inactivity), as indicated by the attenuation of the association between education and SCORE2 in multivariable models.

The biochemical abnormalities observed in this study—including elevated total cholesterol, LDL, and triglycerides, as well as impaired glucose tolerance—represent well-established risk factors for atherosclerosis and cardiovascular morbidity. Consensus statements emphasize that obesity, dyslipidemia, and disturbances in glucose metabolism collectively account for a substantial proportion of excess cardiovascular mortality [41]. Importantly, in our analysis, BMI was significantly associated with lipid and glucose parameters as well as estimated cardiovascular risk, but not with a family history of myocardial infarction or stroke. This suggests that, within this cohort, modifiable factors such as excess body weight and lifestyle behaviours may be stronger determinants of current cardiovascular risk than non-modifiable genetic predisposition. From a public health perspective, this reinforces the potential impact of interventions focused on weight management, smoking cessation, and physical activity promotion, particularly when implemented systematically in primary care.

From a nutritional perspective, several of the abnormalities observed in this study (central adiposity, elevated blood pressure, dyslipidaemia, and impaired fasting glucose) are plausibly shaped by habitual dietary exposures that were captured only at a general level in the CHUK questionnaire. In particular, excessive dietary sodium and a low intake of plant foods are associated with higher blood pressure, whereas diets high in saturated fats and low in unsaturated fats may contribute to higher LDL cholesterol levels. Low HDL-C, which commonly co-occurs with insulin resistance and central adiposity, is also diet-sensitive; dietary patterns richer in unsaturated fats and fibre-rich plant foods, and lower in refined carbohydrates/added sugars, are generally associated with a more favourable HDL/TG profile [8,9,10,12]. At the same time, high intakes of refined carbohydrates and added sugars can promote hypertriglyceridaemia and dysglycaemia through hepatic de novo lipogenesis and reduced insulin sensitivity [9,10,12].

Dietary patterns provide an additional interpretative layer for the clustering of risk factors seen in our sample. Mediterranean-style eating has been associated with improvements in several components of the metabolic syndrome, including waist circumference and fasting glucose, which is relevant given the strong links between central obesity and adverse biochemical profiles observed in this context. Conversely, higher consumption of ultra-processed foods is typically characterized by higher energy density, lower fiber content, and greater amounts of salt, added sugars, and unfavorable fats—features that may jointly promote weight gain and worsen blood pressure and lipid/glucose homeostasis. This could partially explain why participants with multiple behavioural risk factors tended to present a more adverse cardiometabolic profile [13,14].

These considerations have practical implications for PHC. When elevated BMI/waist circumference or abnormal laboratory values are identified during screening, structured dietary assessment (even brief tools focusing on ultra-processed foods, sugar-sweetened beverages, fruit/vegetable intake, whole grains, and the main fat sources) may help to prioritise patients for intensified counselling or referral for medical nutrition therapy, which has demonstrated efficacy in dyslipidaemia management. Importantly, because diet was not quantified in detail in the present study, residual dietary confounding cannot be ruled out; nevertheless, integrating nutrition-focused interventions into routine prevention pathways may enhance the translation of screening results into sustained risk reduction [7,11,12].

A strength of this study is its real-world design in a routine primary care setting, utilizing a nationally standardized NFZ CHUK questionnaire in combination with objective anthropometric and biochemical measurements in a relatively large sample of 506 patients. The comprehensive panel of assessed parameters (BMI, waist circumference, lipid profile, fasting glucose, blood pressure, and SCORE2 risk) allows for an integrated view of risk factor clustering, which is rarely reported in studies based on everyday primary care practice.

A key contribution of this study lies in the multivariable modelling and analysis of risk factor clustering. Logistic regression analysis revealed that older age (≥50 years), male sex, obesity, and current smoking were independent factors associated with high SCORE2 risk (≥5%) after adjustment for other covariates. In numerical terms, age ≥ 50 years was associated with an almost 30-fold increase in the odds of high SCORE2 risk, male sex with a nearly 4-fold increase, obesity (BMI ≥ 30 kg/m^2^) with approximately a 4.5-fold increase, and current smoking with more than a 7-fold increase in risk. In contrast, self-reported physical activity and family history of cardiovascular events were not significant in the fully adjusted model, which may reflect limitations of simple activity measures and the stronger influence of contemporaneous metabolic and behavioural factors. In a second model, age, male sex, and obesity independently predicted the presence of at least one non-optimal biochemical parameter, confirming their central role in shaping the metabolic risk profile. These findings are consistent with contemporary evidence and ESC consensus statements, which identify age, male sex, adiposity, and smoking as core drivers of global cardiovascular risk [41].

The clustering analysis further demonstrated a clear dose–response relationship between the number of coexisting modifiable risk factors and high SCORE2 risk. Only about one in ten patients had no risk factors, while the majority had two or more. The prevalence of high SCORE2 risk rose from just over 1% in patients with 0–1 factor to around 9% in those with 2–3 factors and to more than a quarter in those with 4–5 coexisting factors. This pattern highlights the cumulative nature of cardiovascular risk in everyday primary care populations, underscoring the importance of comprehensive risk assessment rather than focusing on single parameters in isolation. Identifying patients with clustered risk factors may help to prioritise intensive interventions and follow-up in primary care, especially in individuals who are still relatively young but already exhibit an adverse risk factor profile. Although the study was conducted in a single urban primary care centre, the observed clustering of cardiometabolic risk factors and patterns of preventive screening is likely relevant to other urban primary care populations in health care systems with comparable organisation of PHC services. Therefore, our findings may serve as a reference for planning preventive interventions, identifying high-risk groups, and optimising routine screening pathways in similar urban settings.

Overall, the results of this study confirm that BMI, waist circumference, smoking, physical inactivity, and socioeconomic factors are significantly associated with cardiovascular risk markers in primary care patients. These findings align with the international literature and underscore the need for integrated prevention strategies in primary care settings [31,32,33,40,41]. Routine assessment of waist circumference, in addition to BMI, systematic screening of lipid and glucose parameters, early identification of patients with clustered risk factors, promotion of physical activity, smoking cessation, and targeted interventions in high-risk groups should be prioritized in cardiovascular prevention programmes. Future research should include longitudinal follow-up to assess the progression from clustered risk factors to manifest CVD and to evaluate the effectiveness of multifactorial interventions in real-world primary care conditions.

## 5. Limitations of the Study

This study has several limitations that should be considered when interpreting the results. First, it was conducted in a single primary care centre (Medical Center of Primed in Lublin) and included patients who voluntarily agreed to participate in the project. However, the study reflects real-world conditions of a typical urban primary care practice and provides valuable insight into everyday clinical populations.

Because the analyses are cross-sectional and based on a single preventive screening visit, causal inferences cannot be made, and hard cardiovascular endpoints (e.g., myocardial infarction, stroke, cardiovascular mortality) were not available. SCORE2 was therefore used to quantify baseline 10-year risk; future longitudinal studies should evaluate how the identified risk-factor clusters translate into incident cardiovascular events.

Physical activity was assessed using a simplified, dichotomous variable (active vs. inactive). While operational for routine preventive practice, this approach does not capture frequency, intensity, duration, or type of activity, and may have reduced the precision of associations with metabolic outcomes.

Biochemical parameters (lipid profile and fasting glucose) and blood pressure were measured only once, in routine clinical conditions. Although analyses were performed according to current laboratory recommendations, intra-individual variability and temporary factors (e.g., short-term changes in diet, stress, acute conditions) may have influenced the results.

Despite these limitations, the study offers clinically relevant insights into the clustering of modifiable cardiovascular risk factors in a real-world primary care setting. The use of a standardised national questionnaire, combined with objective anthropometric and biochemical measurements, strengthens the reliability of the findings and underscores the need for integrated, multifactorial prevention strategies at this level of care.

## 6. Conclusions

In this real-world primary care population, excess body weight, central obesity, dyslipidaemia, and impaired fasting glucose were common, and together they formed an unfavourable cardiometabolic profile compatible with metabolic syndrome.

Older age (≥50 years), male sex, obesity (BMI ≥ 30 kg/m^2^), and current smoking were the main independent determinants of high 10-year cardiovascular risk (SCORE2 ≥ 5%), while age, sex, and obesity were also associated with the presence of at least one abnormal biochemical parameter.

A clear dose–response relationship was observed between the number of coexisting modifiable risk factors and high SCORE2 risk: the proportion of high-risk patients increased from just over 1% in those with 0–1 factor to more than 25% in those with 4–5 factors. Patients with lower educational attainment were more likely to be classified as high risk, suggesting an important role of socioeconomic determinants.

These findings underline the need for a comprehensive approach to cardiovascular prevention in primary care, including routine assessment of BMI and waist circumference, regular lipid and glucose testing, and systematic use of SCORE2 risk estimation. Particular attention should be given to patients with clustered risk factors and to those who are older, male, obese, and current smokers.

Integrating clinical risk assessment with targeted lifestyle support—such as smoking cessation, dietary counselling, and promotion of physical activity—may improve the effectiveness of preventive activities in PHC. Future longitudinal studies are needed to assess the long-term effects of such multifaceted interventions on cardiovascular outcomes in everyday primary care practice.

## Figures and Tables

**Table 1:** Characteristics of the study population and selected health-promoting and health-risk behaviours.

Characteristic of a Research Group	Categories	N* (*N* = 506)	%	p*
Gender	Woman	187	37	0.006
	Man	319	63	
Age	<50	402	79	0.001
	55–60	88	17	
	>60	16	3	
BMI	underweight	6	1.2	0.3519
	normal weight	284	56	
	overweight	160	31.6	
	obesity class I	43	8.5	
	obesity class II	9	1.8	
	obesity class III	4	0.8	
Waist Woman [cm]	>80	171	34	0.001
	<80	148	29	
Waist Man [cm]	>95	97	19	
	<95	91	18	
Education	higher education	402	79	0.001
	incomplete higher education	7	1.5	
	secondary education	90	18	
	vocational education	7	1.5	
Type of work	white-collar work	93	18	0.002
	manual work	21	4.4	
	unemployed	2	0.4	
	retired/on disability pension	9	1.8	
	other	164	32.4	
Physical activity	yes	246	48.6	0.137
	no	260	51.4	
Smoking	no	417	82	0.041
	since the age of 10	47	9	
	since the age of 15	15	3	
	since the age of 20	18	4	
	since the age of 30	9	2	
Perception of smoking as harmful	yes	331	65	0.045
	no	175	35	

BMI = body mass index; N* = number of participants, p* chi-square test.

**Table 2:** Detailed diagnostic assessment for cardiovascular diseases (CVD).

Biochemicals Parameter	Categories	*N*	%	p*
Total cholesterol [mg/dL]	normal (≤200)	267	53	0.048
	borderline (201–250)	207	41	
	abnormal (>250)	32	6	
LDL [mg/dL]	normal (≤135)	370	73	0.004
	borderline (136–150)	69	13.6	
	abnormal (>150)	67	13.4	
HDL [mg/dL]	minimum 40 men	29	6	0.121
	minimum 50 women	52	10	
	below 40	404	80	
Triglycerides [mg/dL]	normal (≤150)	405	80	0.003
	borderline (151–199)	58	11.5	
	abnormal (>200)	43	8.5	
Glucose [mg/dL]	normal (≤99)	446	88	0.003
	impaired fasting glucose/prediabetes (100–125)	57	11.3	
	diabetes (>125)	3	0.6	
Myocardial infarction/stroke in the family	Yes	33	6.5	0.229
	No	473	93.5	
Cardiovascular risk [%]	None	93	18	0.001
	<1	151	30	
	1–5	217	43	
	5–9	35	7	
	10–15	8	2.5	
	>15	2	0.5	
Blood pressure (BP) [mmHg]	120–129/80–84	288	57	0.001
	130–139/85–89	153	30	
	140/90	65	13	

p*, chi-square test.

**Table 3:** Significance indices in relation to BMI (by chi-square test).

Parameter	Total Cholesterol [mg/dL]	LDL [mg/dL]	HDL [mg/dL]	Triglycerides [mg/dL]	Glucose [mg/dL]	Myocardial Infarction/ Stroke in the Family	Cardiovascular Risk [%]
BMI	0.008	0.001	0.001	0.001	0.002	0.7075	0.001

**Table 4:** Multivariable logistic regression for high cardiovascular risk (SCORE2 ≥ 5%) among primary care patients (*N* = 506).

Parameters	OR (95% CI)	*p*-Value
Male sex (vs. female)	3.89 (1.76–8.61)	<0.001
Age ≥ 50 years (vs. <50 years)	29.02 (11.73–71.79)	<0.001
Obesity (BMI ≥ 30 kg/m^2^)	4.56 (1.80–11.55)	0.001
Current smoking (yes vs. no)	7.16 (3.05–16.82)	<0.001
Physical activity ≥ 1 time/week (yes vs. no)	1.45 (0.67–3.17)	0.346
Family history of MI/stroke (yes vs. no)	0.88 (0.22–3.52)	0.851

Dependent variable: high cardiovascular risk (SCORE2 ≥ 5% vs. <5%); High cardiovascular risk was defined as SCORE2 category 5–9%, 10–14%, or >15%. Model adjusted simultaneously for sex, age category, obesity, current smoking, physical activity, and family history of myocardial infarction or stroke.

**Table 5:** Multivariable logistic regression for any non-optimal biochemical parameter among primary care patients (*N* = 506).

Parameters	OR (95% CI)	*p*-Value
Male sex (vs. female)	2.08 (1.39–3.12)	<0.001
Age ≥ 50 years (vs. <50 years)	3.35 (2.01–5.59)	<0.001
Obesity (BMI ≥ 30 kg/m^2^)	2.16 (1.10–4.28)	0.026
Current smoking (yes vs. no)	0.89 (0.55–1.46)	0.656
Physical activity ≥ 1 time/week (yes vs. no)	0.84 (0.57–1.22)	0.353

Dependent variable: presence of any non-optimal biochemical parameter (total cholesterol, LDL, HDL, triglycerides, or fasting glucose). “Any non-optimal biochemical parameter” was defined as: borderline or high total cholesterol (≥201 mg/dL), borderline or high LDL cholesterol (≥136 mg/dL), low HDL cholesterol (<40 mg/dL), borderline or high triglycerides (≥151 mg/dL), or impaired fasting glucose/diabetes (≥100 mg/dL). Model adjusted for sex, age category, obesity, current smoking, and physical activity.

**Table 6:** Grouped number of risk factors and high SCORE2.

Number of Risk Factors	*N*	*N* with SCORE2 ≥ 5%	% with SCORE2 ≥ 5%
0–1	171	2	1.2%
2–3	260	24	9.2%
4–5	74	19	25.7%

**Table 7:** Education level and high SCORE2 risk (univariate analysis, *N* = 506).

Education Level	*N*	*N* with SCORE2 ≥ 5%	% with SCORE2 ≥ 5%
Less than tertiary	98	17	17.5%
Tertiary education	408	28	6.9%

Chi-square: χ^2^ = 9.70, *p* = 0.0018. Education classified as: lower than tertiary = no higher education (secondary, vocational, incomplete higher education); tertiary education = higher education.

## Data Availability

All data are available in the article.
